# Behind the white coat: Unraveling the patterns of workplace violence in an Italian healthcare setting – An epidemiological exploration

**DOI:** 10.1371/journal.pone.0324545

**Published:** 2025-05-28

**Authors:** Claudio Terranova, Clara Cestonaro, Federico Ferrari, Ludovico Fava, Alessandro Cinquetti, Anna Aprile

**Affiliations:** 1 Department of Cardiac, Thoracic, Vascular Sciences and Public Health, Legal Medicine and Toxicology, University of Padova, Padova, Italy; 2 Department of Neuroscience, Psychiatry, University of Padova, Padova, Italy; The Fourth People's Hospital of Chengdu, CHINA

## Abstract

**Aim:**

In the present study, we aimed to provide an epidemiological and descriptive overview of violence against healthcare workers in an Italian university hospital, presenting and characterizing the risk factors in the department where such events occur and to propose ways to prevent aggressive behaviors.

**Methods:**

We retrospectively analyzed violence against healthcare workers by patients and attendants at an Italian university hospital from 2020 to 2022. Aggressions were documented in anonymous incident reports collected by the hospital’s Clinical Risk Unit. The frequencies and percentages were calculated via a descriptive analysis. Chi-square tests were used to compare the wards with the most aggressions to other wards.

**Results:**

Of the 219 included cases, the aggressors were primarily male patients and the victims female nurses. Most of the aggressions occurred in the psychiatry and emergency department. Among the aggressors, 41.1%, had a psychiatric diagnosis or neurocognitive impairment. Over half the cases involved physical aggression. Compared to other wards, psychiatric wards showed an even distribution of aggressor gender, a higher proportion of male victims, fewer verbal aggressions, and less impact from environmental factors. Notably, female aggressor status (p < 0.001, OR = 8.687) and involvement in physical assaults (p < 0.001, OR = 15.236) were identified as independent risk factors associated with aggression in psychiatric settings.

**Conclusion:**

Our findings align with the literature in that most of the incidents occurred in the psychiatry and emergency medicine departments and involved nurses. However, physical rather than verbal aggression predominated. Notable distribution, risk factor, and qualitative differences were observed between the psychiatric and non-psychiatric services, which warrants further investigation. Our results could be useful in implementing better prevention strategies based on the type of ward.

## Introduction

Violence against healthcare professionals, a widespread global issue with clinical and legal implications [1], according to the National Institute for Occupational Safety and Health (NIOSH) is defined as “any physical assault, threatening behavior, or verbal abuse occurring in the workplace” [2,3]. Violence can be attempted, physical, verbal, or sexual in nature [4]. Within the conceptualization and understanding of aggressive behavior, Walker’s cycle of violence theory [5,6] which was initially devised to elucidate patterns within abusive couple relationships, offers a cyclical model for understanding aggressive behavior. By examining the stages outlined in Walker’s model, healthcare staff can gain valuable insights into the progression of violent behavior and potentially intervene to disrupt its cycle [7].

According to the NIOSH [2], from 2002 to 2013, incidents of workplace violence were considerably more frequent in the healthcare and social assistance sectors compared to other areas [8]. Available data and studies have consistently indicated that the phenomenon affects at least half of healthcare professionals; however, it is widely believed that the available data represent only the tip of the iceberg in a significantly underestimated situation. Underreporting can be explained [9,10] by the widespread perception that aggression is structurally part of professional activity, especially in the nursing sector.

In 2014, Spector [11] conducted a systematic review of data involving approximately 160,000 nurses globally. The findings highlighted that physical violence is more frequent in psychiatric services, emergency departments, and geriatric units. Verbal violence, on the other hand, was reported as involving all services, with a significantly lower incidence in geriatric services. Regarding regional differences, Anglo-Saxon countries report a higher incidence of physical violence and sexual harassment. In contrast, non-physical violence is least frequent in Asia, while the Middle East records the lowest incidence of physical violence but the highest prevalence of verbal aggression. Additionally, in both Asian and Middle Eastern contexts, physical and verbal violence are more often perpetrated by patients’ family members and friends [11]. In contrast, the involvement of family and friends is rare in European and Anglo-Saxon regions, where patients themselves are the main aggressors [11,12]. These data could be linked to the complex sets of values in the two socio-anthropological contexts, namely, the more collectivistic society in Asia, which justifies the joint action of a patient’s relatives, and the individualistic dimension that prevails in Europe [13]. Other socio-anthropological factors that may explain the lower rate of physical violence in the Middle East include the lesser tendency for physical contact between men and women, as men are typically the perpetrators of physical violence [11,14]. Building on these regional differences, the specific hospital settings where violence occurs must also be considered. According to Spector [11], as well as Lim [15], Chakraborty et al. [16], and Azami et al. [8] psychiatric wards and emergency departments are the contexts with the highest risk of aggressions.

Regarding the role of the victims, data from U.S. hospitals reveal that 100% of emergency nurses have encountered verbal abuse, and 82.1% have experienced physical violence [12]. Medical doctors, however, face violent incidents less often than their nursing counterparts [17]. A meta-analysis by Berger et al. published in 2024, which included 75 studies comprising over 139,000 healthcare workers from 32 countries, focused on violence against healthcare professionals in intensive care units. The researchers found an overall median frequency of violence of 51%, and verbal violence was identified as the most common type of violence. Younger age, less work experience, and shift and night work were observed to be risk factors associated with exposure to violence, with the latter likely because of reduced staff. On the other hand, impairment through drugs, miscommunication/distrust, and waiting hours/inflexible visiting hours were included among the patient characteristics reported as associated with violence against healthcare workers [18]. Long wait times, frustration over treatment, and a lack of attention have further been considered as factors potentially causing patient anger and agitation [19]. With regard to patient – physician mistrust, Tucker et al. referred to patients’ perceptions of injustice within the medical context and the lack of humanistic components, which are important for empathy and caregiving, in physician training [20].

Beyond individual and environmental risk factors, the effectiveness of institutional responses to workplace violence must also be considered. A report by the European Agency for Safety and Health at Work (EU-OSHA) [21] highlights the importance of recognizing both physical and verbal aggression, promoting incident reporting, and establishing consistent systems for documenting violence—an area where practices remain uneven, even within the same country. Similarly, collecting standardized and comparable data on this issue remains a challenge in Italy [21]. In 2021, the Italian State Institute INAIL, which protects workers from physical and economic loss resulting from accidents and illnesses, reported that 1,382 incidents of injury related to workplace aggression in the healthcare sector were recorded and recognized - a 322% increase compared to 2005; half of these events had involved nurses [22]. The involvement of nurses in episodes of verbal or physical violence in the Italian context has been confirmed by the National Italian Federation of Orders of Professional Nurses and by studies conducted nationwide [23,24], with the reported prevalence exceeding 90% [23].

Due to the high frequency of aggression toward healthcare professionals, it is essential to strengthen reporting systems and promote staff collaboration. Encouraging the reporting of verbal and physical violence is vital for raising awareness and plays a key role in clinical risk management [25]. The active involvement of healthcare workers in documenting such incidents is fundamental to improving care quality, enhancing the healthcare system, and ensuring the safety of both patients and providers [22]. To analyze the phenomenon, it is therefore essential to consider not only the previously mentioned elements, such as the types of departments and patients, the types of healthcare workers, the gender of the healthcare workers, and the socio-anthropological aspects, but also other possible causal factors. Armed conflicts [26], economic factors [27,28], healthcare system delivery, organizational factors [28], and events such as pandemics [29–31] can lead to increases in violence and should be taken into consideration.

To contribute to the analysis of workplace violence in the healthcare setting, which may exhibit particular characteristics depending on the specific territory being examined, the primary objective of our study was to establish an epidemiological and descriptive overview of violence against healthcare workers in an Italian university hospital. Additionally, we aimed to identify and characterize the risk factors and determinants of aggressive behavior directed at healthcare workers. Based on these findings, we further aimed to recommend actions to enhance the prevention and containment system for this phenomenon.

## Materials and methods

### Population studied and data source

We conducted a retrospective-observational study on aggressions against healthcare workers that occurred at the University Hospital of Padua, a major tertiary care and teaching hospital in Northern Italy. The study period spanned from January 1, 2020, to December 31, 2022. The aggressions were reported by healthcare workers of the University Hospital of Padua themselves using incident reporting forms, which were collected and managed by the hospital’s Clinical Risk Unit. These forms are deliberately and anonymously submitted by the workers to the Clinical Risk Unit to identify factors that may have contributed to the incidents, without attributing blame to the reporting professional. The anonymous information concerning aggression, which excluded details that could identify the patient or the healthcare worker, was recorded in an Excel spreadsheet which was provided to the authors on May 2, 2023.

The Ethics Committee for Clinical Experimentation of Padua acknowledged the study on November 24, 2022 (code 310n/AO/22).

### Inclusion and exclusion criteria

All aggressions committed by patients, visitors, or relatives against healthcare professionals (doctors, nurses, healthcare operators, midwifery staff) were included in the study. Aggressions that occurred outside the examined time frame, were committed by another employee of the University Hospital of Padua, did not involve a healthcare professional, and for which incomplete essential data were supplied for event characterization (e.g., the ward where it occurred, the victim’s role, episode description) were excluded from the study.

### Data collection form

The data were extracted from the forms previously collected by the Clinical Risk Management Unit. After anonymization, the data were captured in an Excel spreadsheet.

The following variables related to (1) the aggressor, (2) the event, and (3) the victim of the aggression were included in the spreadsheet.

(1) Aggressor: role (patient, relatives), sex (male, female), pre-existing psychiatric pathology before the aggression (present/absent).
(2) Event: date expressed in month, day, year, occurrence time divided into time slots (0:00–6:00, 6:00–12:00, 12:00–18:00, 18:00–0:00), weather on the day of the event, type of department, psychiatric or non-psychiatric department, location of the aggression in the department (patient’s bed, outpatient clinic, department corridor, department room, department exit, waiting room), mode of aggression (verbal, verbal and physical, pantoclastic crisis [i.e., destroying surrounding objects], aggression using blunt or sharp objects, sexual aggression, other types of aggression), possible prevention of the event according to the operator, possible factors favoring the event, possible factors mitigating the event, involvement of law enforcement.
(3) Victim: role (nurse, healthcare worker, doctor, other), gender, age group, psychological consequences of the event.

### Statistical analysis

The categorical nominal variables in the data collection form were transformed into numerical values. We conducted a descriptive analysis of the entire sample using the categorical/nominal variables by calculating the frequencies and percentages. Subsequently, the cases that had occurred in the department with more aggressions were compared with the remaining cases using the chi-square test for the nominal variables, and unadjusted odds ratios (OR) were reported.The variables that showed significant differences between the two groups (p < 0.05) were included in a binary logistic regression model, with the location of admission in the department with more aggression as the dependent variable. Adjusted odds ratios (AOR) were derived from this multivariate model. A chi-square test was then used to determine whether there was a significant difference in the modes of aggression between the patients with neurocognitive impairments and those without.

All analyses were performed using IBM SPSS Statistics for Windows, version 28.0 [32].

## Results

**Fig 1:**
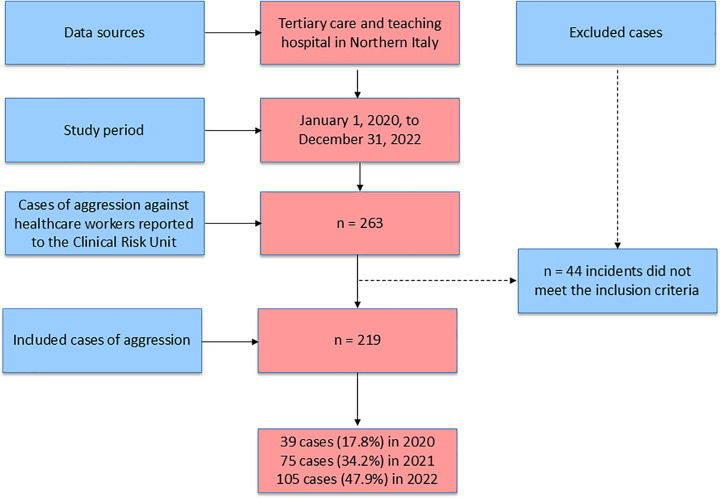
Flowchart of reported cases of aggression against healthcare workers (2020–2022).

During the study period (2020–2022), a total of 263 cases of aggression were reported, of which 219 met the inclusion criteria and were analyzed. Details of the case selection and yearly distribution are shown in Fig 1.

Among the analyzed cases, 78.1% (n = 171) involved patients as the aggressors, while 21.9% (n = 48) involved visitors or relatives of patients. The gender distribution of the aggressors was 66.7% male (n = 146) and 33.3% female (n = 73). With regard to mental health factors, 41.1% (90 out of 219) of the aggressors had a known preexisting psychiatric diagnosis or neurocognitive impairment. The remaining 58.9% (129) did not have a known psychiatric condition. In 24.2% (53 out of 219) of the cases, the healthcare worker was alone at the time of the aggression, while in the remaining 75.8% (166) of the cases, the worker was with others. Notably, 54.3% (119) of the incidents involved aggression toward multiple healthcare workers.

The highest number of aggressions (33.8%, 74 out of 219) occurred in the fourth quarter of the year (i.e., October–December). Lower rates were noted in other quarters: 16.9% (37 cases) in the first quarter, 20.5% (45 cases) in the second quarter, and 28.8% (63 cases) in the third quarter. Regarding the distribution of incidents across the week, over one-third of aggressions (35.2%, 77 out of 219) took place on weekends starting from Fridays, while the remaining 64.8% (142) occurred during the first four days of the week. Specifically, the following events (number and percentage) were reported for each day of the week: Monday 21 (9.6%), Tuesday 43 (19.6%), Wednesday 43 (19.6%), Thursday 35 (16.0%), Friday 34 (15.5%), Saturday 19 (8.7%), and Sunday 24 (11.0%). In terms of timing, 9.1% (20 out of 219) of aggressions occurred between 00:00 and 06:00 am, 27.4% (60) between 06:00 and 12:00, 32% (70) between 12:00 and 18:00, and 31.5% (69) between 18:00 and 00:00. The analysis of the event occurrence time in relation to the phase of the week (weekend or weekday) did not show any significant differences in the distribution of events.

Regarding the influence of weather on the occurrence of aggression, more than half the episodes (52.1%, 114 out of 219) occurred on clear weather days, 31.5% (69) during rain and/or snow, and the remaining 16.4% (36) on cloudy days without precipitation. However, our analysis of key dependent variables—such as modes of aggression, ward type, and the presence of neurocognitive disorders in patients—revealed no statistically significant associations with meteorological conditions, including sunny, cloudy, or rainy weather (p > 0.05).

The departments with the most reported cases were psychiatry (26.9%, 56 out of 219 cases), the surgical departments collectively (15.1%, 33), and the emergency medicine department (13.2%, 29) (refer to Table 1).

**Table 1:** Number of events per ward.

Ward denomination	Number (%)
**Psychiatry**	59 (26.9)
**Surgical Department**	33 (15.1)
**Emergency Department**	29 (13.2)
**Internal Medicine Units**	23 (10.5)
**Intensive Care Unit**	18 (8.2)
**Outpatient Clinics**	13 (5.9)
**Orthopedic Departments**	10 (4.6)
**Child Neuropsychiatry**	10 (4.6)
**Geriatric Departments**	8 (3.7)
**Pediatrics**	6 (2.7)
**Gynecology**	6 (2.7)
**Other Departments**	4 (1.8)

Detailed information on the places of occurrence and modes of aggression is provided in Tables 2 and 3, respectively. Notably, the majority of events took place in the corridor of the ward (25.6%), followed by the waiting room (18.7%). In more than half the cases, the aggression was physical. This data was derived from various elements that described the nature of the aggression. Hence, the utilization of blunt objects (46 cases, 21% of the total aggressions), the co-occurrence of verbal aggression alongside physical aggression (68 cases, 31.1%), the destruction of surrounding objects during pantoclastic crises (6 cases, 2.7%), and the involvement of sharp weapons (2 cases, 0.9%) all contributed to the overall figure (122 cases, or 55.7% of the total aggressions).

**Table 2:** Number of events in relation to the place of occurrence.

Place of Occurrence	Number (%)
**Ward Corridor**	56 (25.6)
**Ward Room**	51 (23.3)
**Waiting Room**	41 (18.7)
**Patient Bed**	34 (15.5)
**Outpatient Room**	34 (15.5)
**Ward Exit**	3 (1.4)

**Table 3:** Type of aggression.

Type of Aggression	Number (%)
**Verbal**	92 (42.0)
**Verbal and Physical**	68 (31.1)
**Contusive Action** ^*^	46 (21.0)
**Pantoclastic Crisis** ^**^	6 (2.7)
**Other**	5 (2.3)
**Use of Sharp Object**	2 (0.9)

Note.

*Contusive action. With this description, reference is made to an aggression toward an operator using a blunt object. Blunt objects (objects or hands and feet) can cause injuries, such as abrasions, bruises, and fractures.

**Pantoclastic crisis. This term is used to describe a situation in which a patient tends to aggressively break any object found in the surrounding environment, even toward healthcare workers trying to contain them.

When queried about potential mitigating factors, 63% (138 out of 219) of workers reported the absence of any factors mitigating the event, whereas the remaining workers cited the following factors: early identification of the aggressor (i.e., noticing potentially risky behavior in the patient) (19 out of 219), luck or chance (i.e., the reporting worker identified a reduction in the consequences of the aggression due to a purely random factor) (17 out of 219), effective surveillance (i.e., the worker indicated that patient surveillance reduced the consequences of the aggression) (21 out of 219), and good work organization (interpreted as a set of actions aimed at containing aggression, including, for example, the transmission of information among colleagues or the involvement of medical staff or family members) (24 out of 219). With respect to prevention, more than half the reports (54.8%, 120) indicated that the event was not preventable, while 16.4% (36) stated it was preventable. A further 28.8% (63) of the respondents indicated that it was “maybe” preventable.

We then evaluated the responses provided by the healthcare workers regarding mitigating factors and the contribution of environmental factors in relation to events that they had indicated were preventable compared to those that were not preventable. The identification of an environmental factor as a possible contributing factor to the event was distributed differently between the two groups (p < 0.001), with 75% of the healthcare workers attributing an environmental factor to a potentially preventable event. No difference in distribution was observed regarding the mitigating factors. Overall, nearly half the healthcare workers (45.2%, 99 out of 219) reported that environmental factors played a role in contributing to the aggression in some capacity. Additionally, in 21.4% (47 out of 219) of the descriptions of aggression, law enforcement intervention was required, highlighting the severity of certain incidents.

Regarding victim roles, nurses were involved in 69.9% (153) of cases, other healthcare workers in 20.1% (44), and medical doctors in 10% (22). Over half (64.8%, 142 out of 219) the victims were women, compared to 35.2% (77) who were men. Twenty-five individuals (11.4% of the sample) were younger than 29 years, 54 (24.7%) were aged between 30 and 39 years, 66 (30.1%) were between 40 and 49 years, 64 (29.2%) were between 50 and 59 years, and 10 individuals (4.7%) were older than 60 years. Regarding the psychological consequences reported by the victims, 128 individuals (56.4%) declared experiencing fear, 96 (43.8%) reported irritation, 89 (40.6%) mentioned anger, 83 (37.9%) expressed a sense of helplessness, 82 (37.4%) felt anxiety, 79 (36%) experienced humiliation, and 8 (3.6%) noted a sense of guilt.

We subsequently compared the events that occurred within psychiatric facilities (the department with the most aggression incidents) with the remaining departments in the study sample. The use of the chi-square test for the nominal variables provided the results reported in Table 4. The odds ratios (OR) reported in Table 4 are unadjusted (crude), as they were derived from bivariate comparisons between psychiatric and non-psychiatric settings, without controlling for potential confounding factors. These exploratory analyses were conducted to identify candidate variables for inclusion in the subsequent multivariate logistic regression model.

**Table 4:** Number of events in psychiatric/ non-psychiatric wards.

Variable	Variable categories	Psychiatric departments N (%) 69 (100)	Non-psychiatric departments N (%) 150 (100)	p-value^*^	OR^**^ (95% CI^***^)
Aggressor’s role	Patient	68 (98.6)	103 (68.7)	<0.001	0.032 (0.004–0.239)
	Visitor/relative	1 (1.4)	47 (31.3)		
Aggressor’s sex	Male	35 (50.7)	111 (74.0)	<0.001	2.765 (1.523–5.020)
	Female	34 (49.3)	39 (26.0)		
Location of incident	Patient’s bed	2 (2.9)	32 (21.3)	<0.001	–
	Outpatient room	13 (18.8)	21 (14)		
	Ward corridor	34 (49.3)	22 (14.7)		
	Ward room	19 (27.5)	32 (21.3)		
	Ward exit	0	3 (2.0)		
	Waiting room	1 (1.4)	40 (26.7)		
Dichotomous diagnosis	Presence of known psychopathological alterations	68 (98.6)	22 (14.7)	<0.001	0.003 (0–0.019)
	Absence of known psychopathological alterations	1 (1.4)	128 (85.3)		
Aggression modality	Physical	63 (91.3)	59 (39.3)	<0.001	16.195 (6.590–39.801)
	Non-physical	6 (8.7)	91 (60.7)		
Attenuating factors	None	37 (53.6)	101 (67.3)	0.017	–
	Early Identification	8 (11.6)	11 (7.3)		
	Luck	6 (8.7)	11 (7.3)		
	Effective Surveillance	4 (5.8)	17 (11.3)		
	Work Organization	14 (20.3)	10 (6.7)		
Working alone	Yes	11 (15.9)	42 (28.0)	0.036	2.051 (0.982–4.283)
	No	58 (84.1)	108 (72.0)		
Favorable environmental factors	Yes	22 (31.9)	77 (51.3)	0.005	2.253 (1.238–4.102)
	No	47 (68.1)	73 (48.7)		
Victim’s sex	Male	41 (59.4)	36 (24.0)	<0.001	0.216 (0.117–0.397)
	Female	28 (40.6)	114 (76.0)		
Age categories	<40	15 (21.7)	64 (42.7)	0.011	–
	40-60	50 (72.5)	80 (53.3)		
	>60	4 (5.8)	6 (4)		

Note.

*The p-values were calculated using Pearson’s chi-square test unless the cell counts were below 5, in which case Fisher’s exact test was employed. The significance level was set at p < 0.05.

**OR = odds ratio

***CI = confidence interval

Following the described analysis, we checked for potential multicollinearity among the variables that were statistically different between the two groups. Based on this analysis, dichotomous diagnosis and the location of the event were excluded from the analysis. The variables that exhibited differences between the two groups were subsequently incorporated into a multivariate binary logistic regression model, with the significance set at 0.05, to explore the factors potentially associated with the reported aggression. The model included independent variables, such as the aggressor’s role, aggressor’s gender, aggression modality, the presence of attenuating factors, environmental factors, victim’s gender, and age categories. The following several significant findings emerged from the model:

Female aggressor status (p < 0.001, OR = 8.687, CI [3.344, 22.567]), involvement in a physical assault (p < 0.001, OR = 15.236, CI [4.642–50.009]), factors contributing to “good working organization” (e.g., risk assessment, sharing of patient characteristics among healthcare providers) (p = 0.006, OR = 5.878, CI [1.656, 20.859]), and the absence of predisposing environmental factors (e.g., confined spaces that pose a higher risk of physical contact) (p = 0.003, OR = 3.711, CI [1.542, 8.934]) were identified as independent risk factors associated with aggression in a psychiatric setting. Conversely, being a female victim proved to be a protective factor against belonging to the group displaying aggression in psychiatry (p < 0.001, OR = 0.112, CI [0.044–0.285]).

All odds ratios (OR) reported in this section are adjusted odds ratios (AOR), derived from the multivariate binary logistic regression model, which controlled for the influence of all other variables included in the analysis.

For a detailed presentation of the logistic regression analysis results, please consult Table 5.

**Table 5:** Factors associated with aggression in the psychiatric department using a multiple logistic regression model.

Variable	p-value	AOR^*^ (95% CI^**^)
**Aggressor’s role** ^***^	0.078	0.117 (0.011 - 1.276)
**Aggressor’s gender** ^****^	<0.001	8.687 (3.344–22.567)
**Aggression modality** ^*****^	<0.001	15.236 (4.642 - 50.009)
No attenuating factors^^^	0.056	
Attenuating factor (early detection of aggressor)	0.155	2.826 (0.676 - 11.816)
Attenuating factor (luck/chance)	0.697	1.328 (0.318 - 5.543)
Attenuating factor (effective surveillance)	0.776	0.802 (0.175 - 3.678)
Attenuating factor (good work organization)	0.006	5.878 (1.656 - 20.859)
**Environmental factors** ^^^^	0.003	3.711 (1.542 - 8.934)
**Victim’s gender** ^^^^^	<0.001	0.112 (0.044 - 0.285)

Note.

*AOR = Adjusted Odds Ratio, obtained through multivariate binary logistic regression^**^ CI = Confidence interval

***Reference category: patient, ^****^ Reference category: male, ^*****^ Reference category: non-physical aggression

^Reference category: no attenuating factor, ^^^^ Reference category: absence of environmental factors, ^^^^^ Reference category: male

In addition to the logistic regression analysis, a chi-square test was employed to perform a distinct comparison between patients with documented neurocognitive/psychiatric impairments and those without such diagnoses. This analysis aimed to explore whether the occurrence of aggression significantly differed between these two groups. The results indicated notable variations in aggression-related factors, such as the modes of aggression (p < 0.001), the gender of the aggressor (p = 0.029), the location of the event (p < 0.001), working with other colleagues (p = 0.021), the impact of environmental factors (p = 0.012), and the gender of the victim (p < 0.001). This secondary chi-square analysis was conducted separately to provide further insight into group-specific differences that are not the primary focus of the logistic regression analysis. It is important to note that patients affected by psychiatric or neurocognitive impairments were not necessarily hospitalized in a psychiatric department, as these diagnoses may be present across various hospital units.

## Discussion

Through the present study, we aimed to contribute to the analysis of the phenomenon of aggressions against healthcare workers by providing an epidemiological and descriptive overview of this issue in an Italian university hospital. The methodology employed is innovative in terms of the variables collected, the focus on both the perpetrator and the victim of the aggression, and the attention to the potential risk factors. The data source, represented by incident reporting forms, holds the potential to achieve a higher adherence rate compared to questionnaires characterized by variable response rates [33–36]. Importantly, the data were not influenced by variables that come into play during the administration of questionnaires, such as emphasis on the study’s purpose or presentation methods. Moreover, the incident reporting forms are deliberately and anonymously transmitted from the worker to the hospital’s Clinical Risk Unit, to identify the factors that may have contributed to the occurrence of the event without the possibility of attributing blame to the professional who forwarded the report, which thereby encourages incident reporting.

The first important finding that emerged from our study was the increase in the number of reports during the study period. Such an increase is difficult to interpret. On one hand, in Italy in August 2020, a law was enacted that aimed to ensure more severe punishment would be meted out for aggressions against healthcare workers. This law (Law 113 of August 14, 2020), which reignited public debate on the issue of violence and the protection of healthcare professionals, may have increased the importance of reporting. The creation of a culture that highlights the importance of incident reporting may also have played a role [37]. On the other hand, it is also true that the years 2020–2021 describe a period burdened by a pandemic health emergency, which may have influenced the number of events and the time available to professionals to report episodes. The relative increase in the number of events has been highlighted by other authors [38], who despite significant reductions in the numbers of patients accessing the hospital, recorded an unchanged number of weekly incidents in the emergency department during the pandemic period.

Consistent with previous studies, our analysis indicates that the majority of aggressions were committed by patients rather than visitors and/or relatives/friends [33,39] and that the aggressors were predominantly men [1,12,16,23,33,40,41]. Individual risk factors as well as the socio-anthropological context may explain the higher rate of aggressions by patients rather than patients’ relatives in the referenced setting. According to the literature, patients’ aggressive behaviors can be attributed to somatic or mental health disorders, including substance-related disorders [42], or a reaction to conflict situations; they may stem from fear or frustration or be related to pain or discomfort [43,44]. The possibility of access to the wards by family and friends, especially during the COVID19 pandemic period, could have impacted the results of the study. During the study period, in fact, the World Health Organization published a guide for the prevention and control of infections in healthcare facilities, which included limits to access into facilities by patients’ relatives both to protect them from infection and to reduce the introduction of the virus [45]. The visiting restrictions during the pandemic were shown to have increased mental health problems and to have caused distress and worry among patients and families [46]. The National Nurses United Survey, in late 2020, recorded an increase in workplace violence during the pandemic. This violence was attributed by the participants to the shortage of COVID-19 personnel, changes in the patient population, and restrictions on visitors. Similarly, Spatari et al. conducted a study on reports of violence against healthcare workers from 2018 to June 2023 in a North-Western Italian city: their analysis found differences across the pandemic phases regarding incidents’ color codes, professional groups involved, and locations of the aggression. Moreover, a different ratio of known to unknown aggressors was found, with a greater number of known aggressors during the pandemic phase [47].

When considering the timing of aggression incidents across all wards collectively, our study revealed a higher frequency of aggressions during the afternoon and evening periods. This pattern aligns with findings from previous research [36,41] and implies a potential correlation with shift changes [1] or a decrease in staffing levels during the evening hours. Interestingly, we observed the lowest rates of aggression during the night, which is in contrast to findings in the literature. Studies indicate that night shifts are associated with a high risk of exposure to violence, and nurses who work night shifts are more likely to report physical and non-physical abuse [48]. However, in a study conducted in the emergency department ofa smaller hospital in Italy, Zaboli et al. noted that between 2022 and mid-2023, 91 incidents of violence were carried out against hospital personnel and that those for which complete information was available had occurred to a similar extent during the day (08:00–20:00) and at night (20:00–08:00) (48.2% vs. 51.8%) [23]. The division into different time slots did not allow for a full comparison with the results of this study. Nevertheless, we found that 35.2% of aggressions took place over weekends, and 64.8% occurred during the first four days of the week, which is similar to the results of Zaboli et al., who observed 37.4% and 62.6%, respectively, for the same time periods [23]. The findings of our study could guide organizational changes, particularly in emergency departments where substance abuse and intoxication admissions are more prevalent in the evenings.

Regarding weather conditions, our analysis did not confirm a correlation between meteorological factors and aggression, including mode of aggression, patient types, and ward types. This contrasts with some literature suggesting an association, particularly in psychiatric patient [49]. The lack of correlation could be related to the type of weather conditions collected. For example, atmospheric pressure, temperature, season, and humidity are factors that were not collected in our study but which could influence the behavior of individuals who are vulnerable to meteorological variables [50].

Our numerical data regarding the majority of aggressions occurring in psychiatric and emergency medicine settings confirms the findings in the literature [12,16,33,35], including those derived from meta-analytic studies [16,40,51,52]. The unexpectedly high incidence of aggression in surgical wards should be viewed considering the absence of data normalization based on patient volume. These findings may reflect not only statistical variations but also the nature of surgical procedures. For instance, the elevated expectations of patients and their families regarding surgical outcomes might increase the risk of conflict when clinical progress does not meet those expectations. Similar dynamics likely contribute to the higher frequency of compensation claims in surgical fields. As for data from psychiatric wards and emergency departments, direct comparisons with other studies remain difficult due to methodological differences.Unlike some studies [33,35], where the data source consisted of specific questionnaires given to staff who provided a single service and/or belonged to a particular professional category, we relied on data covering the entire hospital and which were collected through incident reporting.

When considering mixed aggressions (both verbal and physical), incidents involving contusive actions, those associated with the improper use of sharp objects, and pantoclastic crises in our study, physically characterized aggressions comprised slightly more than half the reported incidents, followed by exclusively verbal aggression. This finding contrasts with those of two significant meta-analyses in which verbal aggression predominated [16,40]. Also, this finding contrasts with an Italian multicenter study of La Torre et al. which collected the results of a digitalized self-compilated questionnaire, open between 2018 and 2020. That study found that a total of 366 healthcare workers, corresponding to 10% of the sample (3659 healthcare workers) were victims of physical aggression at work in the last 12 months, whereas a total of 1723 (47.1%) had declared to be a victim of verbal aggression at work in the last 12 months [53]. However, these differences should be interpreted cautiously due to variations in settings and methodology. This factor, in particular, could lead to a tendency to report more cases of physical than verbal aggression. Moreover, these data may vary significantly across departments, as individual units differ in their sensitivity to aggression toward healthcare workers. Verbal aggression, in particular, is often underreported, possibly because it lacks visible physical consequences for the victim. Furthermore, physical aggression is often the expression of inadequately contained verbal aggression [7,9].

Our study revealed that in 44 cases (21.4%), an intervention from law enforcement was necessary due to the severity of the aggression. These data are intriguing and certainly merit further investigation, especially considering that enactment of Law 113/2020 in Italy during the summer of 2020 mandates that all injuries to healthcare professionals be reported to judicial authorities regardless of whether a complaint is made by the victim.

In line with previous literature, we found that the majority of victims were women and belonged to the nursing profession, followed by other healthcare workers and then physicians [12,34,40]. This finding could be explained by the higher likelihood of nurses encountering aggressive behaviors because of the time spent directly caring for patients [54] but could also depend on the perceived vulnerability of the victim. Although consistent with the literature, this data should be considered cautiously, as no normalization of the data concerning the staff composition was performed, and the proportion of female nursing staff in Italy is 76.5% [55].

Fear was the emotion most frequently reported by the healthcare workers in our study, followed in order by aggression and irritation and anger. A similar view was presented by Mueller and Tschan [56]. These emotional reactions, which affect all victims, influence and alter the work life and satisfaction of the healthcare worker, with long-term impacts on the entire department [12,44].

The comparison between psychiatric wards and other departments in the present study highlighted that the majority of aggressions are carried out by patients. These data are understandable given the increased control over access and visits in facilities dedicated to psychiatric patients. The gender distribution of aggressors in psychiatry showed a homogeneous pattern between female and male aggressors, which is consistent with other studies [57,58]. The reliability of reports concerning the gender of the aggressor in psychiatry has, however, been debated by Hiday [59], who posited that the data may stem from a methodological artifact because when verbal and physical violence are considered separately, a greater gender difference emerges, with men showing more physical aggression and women more verbal aggression. This observation seems more consistent in the overall assessment of reports, while for the psychiatric wards, this principle appears to weaken [60,61].

Moreover, in psychiatric wards, reducing criminogenesis to a single factor (gender) loses theoretical robustness, especially when considering neurocognitive impairments. A possible explanation could be that aggressive behaviors in psychiatric patients are more closely associated with intermediate variables, such as substance use, stress, personal history, previous crimes, childhood abuse, and personality, than sociodemographic characteristics [60,61]. The data in the present study also showed higher incidence of physical versus verbal aggression in the psychiatric wards, which could be explained by specific factors, like closed wards, involuntary admissions, and patient psychopathology, which may strongly trigger violence. Prior studies [60,61] have linked increased violence to factors such as involuntary admissions, heightened emotional expression, manic symptoms, and sleep–wake disruptions. These elements may help explain the differing victim profiles observed, with a higher proportion of male victims in psychiatric wards compared to other hospital departments, where female victims are more common. Nonetheless, the gender disparity in psychiatry is less marked than in other areas of healthcare. In the psychiatric wards, environmental factors facilitating or precipitating aggression were observed in 31.9% of cases, while in other services, this figure rose to 51.3%. This may reflect provider perceptions that violence by psychiatric inpatients arises more from intrinsic conditions than extrinsic factors (e.g., emergency room waits). However, it would be inaccurate to deny environmental factors even for psychiatric patients’ violence, as that would imply psychiatric illness alone drives these acts.

In the psychiatry department, less than half the incident reports mentioned factors that mitigated the event severity, while nearly two-thirds of reports in other services included such factors. “Good work organization” was most frequently cited as mitigating psychiatric aggressions. Compared to other healthcare departments, this factor is therefore considered relevant in terms of containing the consequences of violent incidents in these wards. According to healthcare professionals working in psychiatry, it can contain the consequences of aggressive events, likely through risk assessments [58,60] and the sharing of patient characteristics among healthcare workers. The widely recognized higher risk of aggression in psychiatry may translate into greater preparedness and organization, as managing such events nearly weekly requires this.

### Implications of the study results for prevention strategies

The study revealed that incidents of aggression were predominantly concentrated in high-stress hospital units, emphasizing the need for proactive prevention strategies.

In 2016, Philips [12] highlighted that the majority of studies on workplace violence aimed to quantify this phenomenon, whereas few described research on experimental methods for its prevention. Raising awareness among staff and users through visible initiatives, such as posters, along with detailed legal and health impact information, has been deemed to play a preventive role [12]. In this context, the Italian Ministry of Health launched a 2023 campaign to raise public awareness about violence against healthcare workers, highlight its severity, promote positive attitudes toward medical staff, and restore trust between the public and healthcare professionals. According to Arnetz et al. [44], since the catalysts for violence may stem from individual factors, or factors that influence the relationship between patients and healthcare professionals during the care process, in the work environment, or in the broader organization (hospital), the identification of risk factors at each of these levels can facilitate the design of specific interventions.

One such intervention is comprehensive staff training [62]. Professionals must be aware of the risk of being verbally or physically assaulted by patients and should be trained to recognize violent patients and situations that could lead to episodes of violence early. This is especially important in psychiatric units [63]. Where necessary, it is recommended to ensure that two different healthcare professionals are present when interacting with patients and relatives. The use of simulations and scenarios that prepare healthcare professionals for situations of violence has been advocated. In addition, each member of the healthcare team should be aware of the response options before an aggression occurs and should be able to quickly select the most appropriate one when facing such a situation [64]. De-escalation has frequently been recommended as the first-line intervention when aggression is imminent [65]. However, while de-escalation is often effective in stopping a sequence of conflicts in acute healthcare settings, cases with a history of violence can be especially challenging [66].

Stricter legal penalties for violence against healthcare workers—recently introduced in Italy—may help reduce such incidents. However, legal measures alone are insufficient. Clear protocols for managing violence, especially in high-risk settings (e.g., emergency services and psychiatric wards) are crucial. Healthcare workers must also be encouraged to report all incidents by being given proper tools (e.g., incident reporting systems) and guaranteed easy access to psychological and legal support.

### Study limitations

A key limitation of the present study is the voluntariness of the incident reports analyzed, as this likely resulted in events being underestimated or eventually introduced reporting bias [44]. This data source could thus have been influenced by information bias as well as health professionals selection bias. Individuals may be more inclined to report certain types of incidents over others (e.g., physical aggression compared to verbal aggression), which could have led to an underrepresentation of less severe incidents and those perceived as more commonplace.

To address these limitations, future research should explore methods to mitigate potential biases inherent in incident reporting data perhaps through the implementation of standardized reporting protocols or complementary methodologies. Additionaly, missing variables related to the aggressor and the healthcare professional victims in the data collection sheet also limited the interpretability of the results. In psychiatry, a lack of data on hospitalization type (voluntary or involuntary), type of medications, medication adherence, substance use disorders and intoxication episodes limits the interpretation of patient-on-staff aggression [60,61].

### Future perspectives

To develop appropriate prevention measures, it would be useful to trace triage codes and to link them to aggressions that occur in emergency departments [12,67] as well as to collect information about victims’ months/years of work experience in the department where the aggression occurred. Moreover, to develop strategies aimed at mitigating the consequences of aggressions, it would be useful to collect additional information concerning their impact on victims [44,64]. Finally, the analysis of data related to potential civil and criminal cases linked to aggressions against healthcare workers could provide alternative perspectives to those of clinical risk.

## Conclusions

The data from the University Hospital of Padua, which identified a higher number of violent incidents in psychiatric wards and emergency departments, are aligned with the findings in the international literature. Similarly, in line with previous research, we found that predominantly nursing staff is involved. Notwithstanding, in contrast to other studies, physical actions were prevalent over purely verbal ones in the Padua context. Furthermore, we noted differences in the distribution, risk factors, and qualitative aspects of violence between the psychiatric and non-psychiatric services. These differences certainly demand further investigation but provide valuable insights for interpreting the phenomenon of violence against healthcare workers.

## Supporting information

S1 Data Workplace violence dataset. Anonymized dataset used for the epidemiological analysis of workplace violence in an Italian healthcare setting.
(SAV)
